# Rutin-Loaded Solid Lipid Nanoparticles: Characterization and In Vitro Evaluation

**DOI:** 10.3390/molecules26041039

**Published:** 2021-02-16

**Authors:** Federica De Gaetano, Maria Chiara Cristiano, Valentina Venuti, Vincenza Crupi, Domenico Majolino, Giuseppe Paladini, Giuseppe Acri, Barbara Testagrossa, Alessia Irrera, Donatella Paolino, Silvana Tommasini, Cinzia Anna Ventura, Rosanna Stancanelli

**Affiliations:** 1 Dipartimento di Scienze Chimiche, Biologiche, Farmaceutiche e Ambientali, Università degli Studi di Messina, Viale Ferdinando Stagno D’Alcontres 31, 98166 Messina, Italy; fedegaetano@unime.it (F.D.G.); vcrupi@unime.it (V.C.); stommasini@unime.it (S.T.); caventura@unime.it (C.A.V.); 2 Dipartimento di Medicina Sperimentale e Clinica, Università degli Studi di Catanzaro “Magna Græcia”, Campus Universitario “S. Venuta”, Viale S. Venuta, 88100 Catanzaro, Italy; mchiara.cristiano@unicz.it (M.C.C.); paolino@unicz.it (D.P.); 3 Dipartimento di Scienze Matematiche e Informatiche, Scienze Fisiche e Scienze della Terra, Università degli Studi di Messina, Viale Ferdinando Stagno D’Alcontres 31, 98166 Messina, Italy; dmajolino@unime.it (D.M.); gpaladini@unime.it (G.P.); 4 Dipartimento di Scienze Biomediche, Odontoiatriche, e delle Immagini Morfologiche e Funzionali, Università degli Studi di Messina, c/o A.O.U. Policlinico “G. Martino” Via Consolare Valeria 1, 98125 Messina, Italy; gacri@unime.it (G.A.); btestagrossa@unime.it (B.T.); 5 CNR-IPCF Istituto per i Processi Chimico Fisici, Viale Ferdinando Stagno D’Alcontres 37, 98158 Messina, Italy; irrera@ipcf.cnr.it

**Keywords:** rutin, solid lipid nanoparticles, Raman spectroscopy, SEM, antioxidant assay, U373 culture cells

## Abstract

This study was aimed at preparing and characterizing solid lipid nanoparticles loading rutin (RT-SLNs) for the treatment of oxidative stress-induced diseases. Phospholipon 80H^®^ as a solid lipid and Polysorbate 80 as surfactant were used for the SLNs preparation, using the solvent emulsification/diffusion method. We obtained spherical RT-SLNs with low sizes, ranging from 40 to 60 nm (hydrodynamic radius) for the SLNs prepared starting from 2% and 5% (*w/w*) theoretical amount. All prepared formulations showed negative zeta-potential values. RT was efficiently encapsulated within SLNs, obtaining high encapsulation efficiency and drug content percentages, particularly for SLNs prepared with a 5% theoretical amount of RT. In vitro release profiles and analysis of the obtained data applying different kinetic models revealed Fickian diffusion as the main mechanism of RT release from the SLNs. The morphology of RT-SLNs was characterized by scanning electron microscopy (SEM), whereas the interactions between RT and the lipid matrix were investigated by Raman spectroscopy, evidencing spectral modifications of characteristic bands of RT due to the establishment of new interactions. Finally, antioxidant activity assay on human glioblastoma astrocytoma (U373) culture cells showed a dose-dependent activity for RT-SLNs, particularly at the highest assayed dose (50 μM), whereas the free drug showed the lesser activity.

## 1. Introduction

In recent years, various drug delivery systems have been developed with the aim of overcoming the limitations that represent the failure of conventional pharmaceutical dosage forms, such as the poor solubility of the drug in biological fluids or the insufficient plasmatic concentration of drug due to poor absorption, rapid metabolism, and elimination. Among them, colloidal systems formed by polymeric or lipidic substances, such as nanoparticles, nanoemulsions, and liposomes, have aroused most of the interest in pharmaceutical technology [1,2]. In particular, in the early 1990s, Müller [3], Gasco [4], and Westesen [5] focused the attention on a new class of colloidal particles composed of lipids, i.e., solid lipid nanoparticles (SLNs), presenting a dimensional range between 50 and 1000 nm.

If compared to traditional colloidal carriers, SLNs exhibit great potential as suitable drug delivery systems [6,7,8,9] thanks to their low toxicity, large surface area, prolonged drug release, superior cellular uptake, as well as capability to improve solubility and the bioavailability of drugs [10,11]. Furthermore, the hydrophobic nature of lipids makes these systems excellent carriers especially for lipophilic drugs. Finally, compared to polymeric nanoparticles, SLNs have the advantage of being biocompatible and biodegradable [12,13,14,15,16,17]. 

As seen by the increasing number of published papers [18,19,20,21,22,23,24,25], the use of natural active ingredients is becoming the focus of much interest in nutraceutical, cosmetic, pharmaceutical, and medical research. The main reason is their antioxidant, anti-inflammatory, anti-mutagenic anti-cancer, anti-Alzheimer, anti-arthritic, and anti-diabetic activity, which makes them indispensable components in numerous applications. In particular, flavonoids as antioxidant agents, free radical scavengers, and singlet oxygen quenchers have been, and still are, widely investigated [26,27,28,29,30]. Among them, rutin (3,3’,4’,5,7-pentahydroxyflavone-3-rhamnoglucoside, RT), is present in many medicinal herbs, fruits, vegetables, and other plants and is considered beneficial due to its potential protective role for multiple diseases related to oxidative stress [31,32,33]. Despite having high antioxidant activities tested in vitro on different culture cells [34], RT shows in vivo low bioavailability [35] probably related to its chemical structure. It seems that glycosilation in the C3 position plays an important role in reducing bioavailability [36]. Furthermore, the low water solubility of RT should be considered [35]. To overcome these problems, different delivery systems for RT were studied [37], such as complexation with β-cyclodextrin [38] or phospholipids [39], encapsulation into polymeric nanoparticles [40,41], or nanoemulsions [42]. In this way, high anti-inflammatory, anti-cancer, and antioxidant activities were observed through different methods of administration [43,44,45]. Furthermore, delivery systems can be designed to improve the biodistribution of RT in the brain for the treatment of neurodegenerative diseases induced by oxidative stress [46]. These systems could be administered intranasally, representing the nose, through the olfactory and trigeminal nerves, a direct access to the brain, so bypassing the blood–brain barrier [47].

Surface charge and particle sizes of SLNs are crucial in nose-to-brain targeting [48]. In particular, the transport through the human olfactory axons can be affected by particle sizes. These last ones have a typical diameter of 100–700 nm [49], which in turn suggests that only particles within such dimensions will be able to be transferred along this pathway. However, nanoparticles of very small sizes (20 nm) could produce damage effects on excised porcine olfactory epithelium [50]. 

In this study, we realized SLNs formulations loading RT (RT-SLNs) intended for the treatment of neurodegenerative diseases induced by oxidative stress through intranasal administration. RT-SLNs were prepared by a solvent emulsification/diffusion method using Phospholipon 80H^®^ as solid lipid and Polysorbate 80 as surfactant. They were characterized as far as morphology and technological properties (sizes, zeta potential, and encapsulation parameters) are concerned. In addition, Raman investigation was performed in order to get deeper information on the interactions involved in the formation of RT-SLNs. Release profiles of RT from SLNs were evaluated at pH 7.4, and different kinetic models were applied to the obtained data in order to evaluate the rate and mechanism of RT release. Finally, antioxidant activity of the RT-SLNs, in comparison with free RT, was performed in vitro on human glioblastoma astrocytoma (U373) cells.

## 2. Results and Discussion

### 2.1. Technological Characterization of RT-SLNs and In Vitro RT Release Study

Four RT-SLNs samples with different theoretical amount of RT (2%, 5%, 8%, and 10%, *w*/*w*) were prepared, and their size (hydrodynamic radius, R_H_), polydispersity index (PDI), and zeta potential (ζ) were determined. As shown in Table 1, all formulations were obtained with very high yields (%) and all are in the nanometric range, even if the sizes increased as the theoretical amount of the drug increases. This could be attributed to the increase in the drug content present in the emulsion nanodroplets. As a matter of fact, since only a fixed amount of drug can be incorporated in a given amount of lipid, the increase of initial drug concentration requires an increase in the quantity of lipid in the nanodroplets, which will give rise to larger SLNs. Furthermore, a further increase in the amount of drug could cause an increase in the viscosity of the dispersed phase, resulting in the formation of larger particles [51,52].

Formulations prepared with 8% and 10% theoretical amount of RT exhibited also clusters of nanoaggregates, showing low homogeneity. Optimum sizes of 40 and 60 nm were observed for the samples prepared starting from 2% and 5% of RT theoretical amount, respectively. Both formulations exhibited slightly negative ζ values, thus requiring the freeze-dried process to guarantee the physical stability of the nanoparticles. The re-dispersion of the RT-SLNs freeze-dried powder did not produce significant changes of sizes and ζ values nor particles aggregation (data not shown), making these formulations suitable for the proposed therapeutical objective. Considering their low sizes and high homogeneity, the encapsulation parameters were determined for these formulations only. 

The encapsulation efficiency (E.E. (%)) was indirectly determined on the pellets obtained from the first centrifugation of the colloidal suspensions. During the preparation of RT-SLNs, the evaporation of the organic solvent caused the precipitation of large, not interacting aggregates of the excess of lipid, and of the excess of drug not encapsulated into the nanocarriers. They were separated from SLNs colloidal suspensions by centrifugation. By treating the obtained pellets with cold ethanol, RT only was solubilized, whereas the lipids remain undissolved. After that, the alcoholic solutions were filtered and analyzed by UV-Vis spectrophotometer.

As shown in Table 1, high E.E. (%) values were obtained for both formulations, demonstrating the affinity of RT with the used lipid. By increasing the drug concentration from 2% to 5%, the drug content percentage (D.C. (%)) was enhanced.

**Figure 1:**
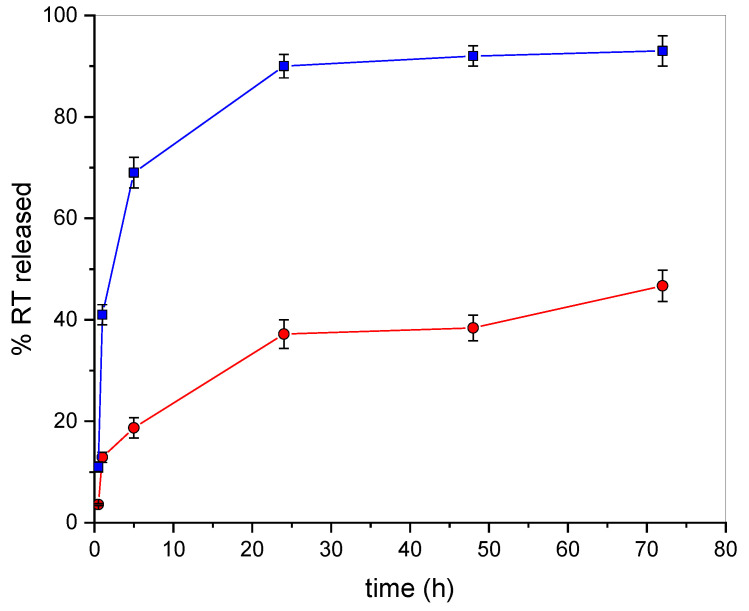
In vitro release profiles of free RT (blue squares) and RT-SLNs (red circles).

Based on the good values obtained for the encapsulation parameters and the small particles size with the absence of clusters, the sample prepared starting from 5% theoretical amount of RT was selected as optimum formulation for a successive in vitro release study, whose results are reported in Figure 1. The drug release of RT from SLNs was investigated by the dialysis of RT-SLNs suspensions using a mixture of phosphate buffer solution (PBS, pH 7.4) and ethanol (15%) as dialysis medium. In this way, the solubilization of RT in the dialysis medium was insured, and sink conditions were maintained. As can be seen from an inspection of the figure, free RT quantitatively passes the membrane within 24 h. RT-SLNs showed an appealing release profile, being able to produce a sustained release of the drug over 72 h. The very low burst effect (about 12%) observed in the first 3 h of the experiment evidenced a low amount of drug on the SLNs surface, suggesting a homogeneous distribution of RT within the lipid matrix. This occurrence, together with the presence of interactions between RT and SLNs, as demonstrated by Raman spectroscopy, could justify the prolonged release of RT from SLNs [53].

As mentioned in the Materials and Methods section, the release data were treated according to zero-order, first-order, Higuchi, and Korsmeyer–Peppas equation models [54]. The release kinetics and the mechanism of RT release from SLNs were evaluated, and the corresponding parameters are reported in Table 2. The transport exponent (n), calculated from the Korsmayer–Peppas model, describes the mechanism according to which the drug loaded into a matrix is released [54].

The model that best fitted with the release data of RT was the Higuchi model, as indicated by the highest value of the regression coefficient (R^2^). Based on this model, the release process of RT from the insoluble matrix of SLNs [55,56] depends on the square root of time, according to a Fickian diffusion [57]. In particular, a value of the exponent n equal to 0.43 is obtained in the case of a Fickian diffusion release mechanism for spherical systems [58].

### 2.2. Scanning Electron Microscopy (SEM)

**Figure 2:**
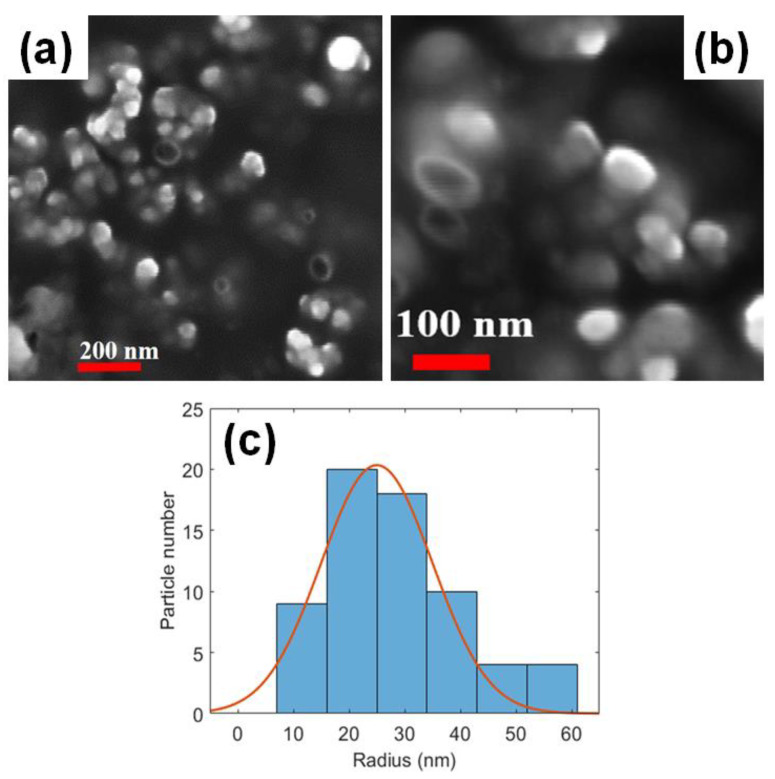
SEM images of RT-SLNs at different magnification (**a**,**b**), together with the Gaussian distribution of the radius peaked at 25 ± 10 nm (**c**).

In Figure 2a,b, the SEM images of RT-SLNs (5% theoretical amount) at different magnifications (as visible by different markers) are reported. The presence of the SLNs is clearly visible as white nanoparticles. 

The solid-state surface morphology in the SEM images showed a three-dimensional network of spherical SLNs with nanometric size and smooth surface. 

By SEM analyses, the radius of nanoparticles was quantified, as described in the Materials and Methods section. We found a radius distribution in size from 10 to 60 nm but clearly peaked at an average of about 25 ± 10 nm (considering the ± σ of the Gaussian distribution).

It is worth noting that the radius of nanoparticles reported in Figure 2 appears significantly different with respect to R_H_ as obtained by dynamic light scattering (DLS). This discrepancy can be justified taking into account the different factors that should be considered when sizes of nanoparticles are determined by different techniques, such as SEM and DLS. First of all, it is worth remarking that the hydrodynamic radius, as obtained by DLS, is an “apparent” radius (conversely to the radius as directly measured by SEM), which reflects the apparent size adopted by the solvated molecule. On the other side, SEM measures sizes of freeze-dried powders whose diameters may be affected by shrinking during sample drying. In addition, considering the negative charge density revealed for our RT-SLNs particles, that should increase the hydration of the particles, a difference between R_H_ and radius obtained by SEM images is expected. Finally, if compared to SEM, DLS probes a larger number of particles that is several orders of magnitude greater, so achieving better statistics.

### 2.3. Raman Spectroscopy

Raman investigation was performed in order to get deeper information on the interactions involved in the formation of RT-SLNs, based on the significant spectral modifications induced on the characteristic vibrational bands of free RT when loaded in SLNs [59,60].

**Figure 3:**
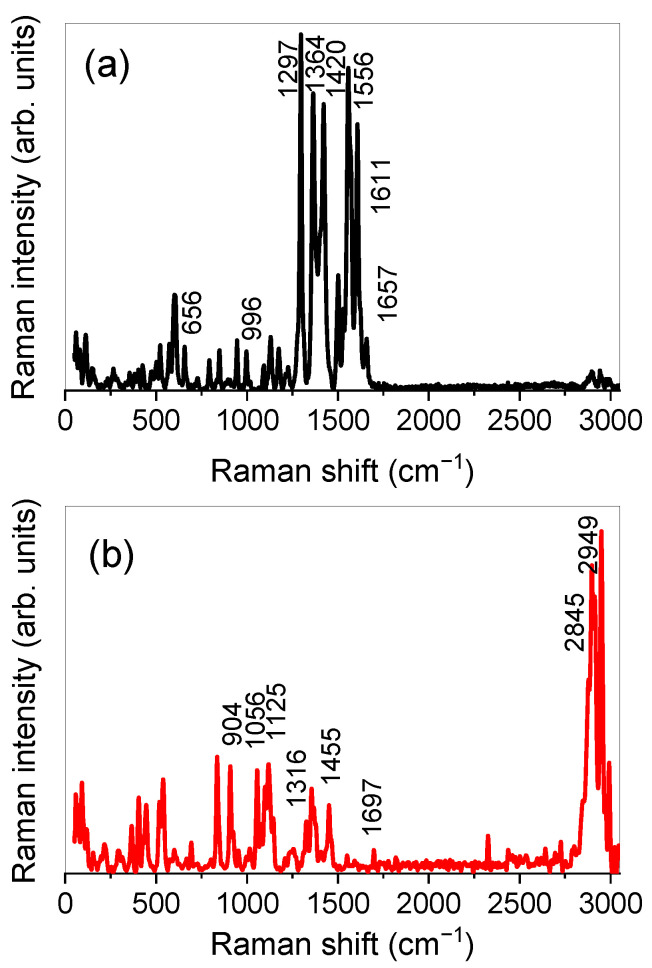
Experimental Raman spectrum of RT (**a**) and SLNs (**b**).

The experimental Raman spectrum of RT is reported in Figure 3a. Since the spectrum, as can be seen, is very composite, only some of the main bands will be discussed.

In particular, the peak at ≈656 cm^−1^ can be associated with deformation vibrations of the benzene, heterocyclic, and mannopyranoside rings. The peak at ≈996 cm^−1^ is ascribed to C-C stretching vibrations of the mannopyranoside and glucopyranoside rings. Going on, the peak at ≈1297 cm^−1^ is due to C–O and C–C stretching vibrations of the heterocyclic ring, the one at ≈1364 cm^−1^ can be mainly ascribed to C–O stretching vibrations of the dihydroxyphenyl ring, while the one at ≈1420 cm^−1^ can be mainly ascribed to C–C stretching vibrations of the benzene ring and the heterocyclic ring. The peak at ≈1556 cm^−1^ is due to C=C stretching vibrations of the benzene and dihydroxyphenyl rings, the one at ≈1611 cm^−1^ is due to C=C stretching vibrations of the benzene, heterocyclic, and dihydroxyphenyl rings and, finally, the peak at ≈1657 cm^−1^ is associated with the C=O stretching vibrations of the heterocyclic ring.

Figure 3b shows the experimental Raman spectrum of SLNs, together with the main vibrational features, detected at ≈904 cm^−1^ (CH_3_ rocking), ≈1056 cm^−1^ (C–C asymmetric stretching), ≈1125 cm^−1^ (C-C symmetric stretching), ≈1316 cm^−1^ (CH_2_ twisting), ≈1455 cm^−1^ (CH_2_ scissoring), ≈1697 cm^−1^ (C=O stretching), ≈2845 cm^−1^ (CH_2_ symmetric stretching), and ≈2949 cm^−1^ (CH_2_ asymmetric stretching).

We decided to focus our attention on the spectral regions extending from 1500 to 1710 cm^−1^ and from 1220 to 1330 cm^−1^ that, being free of vibrational contributions coming from SLNs, allow directly highlighting any changes in the vibrational bands of the RT as a consequence of the entrapment of the drug in the nanoparticles.

**Figure 4:**
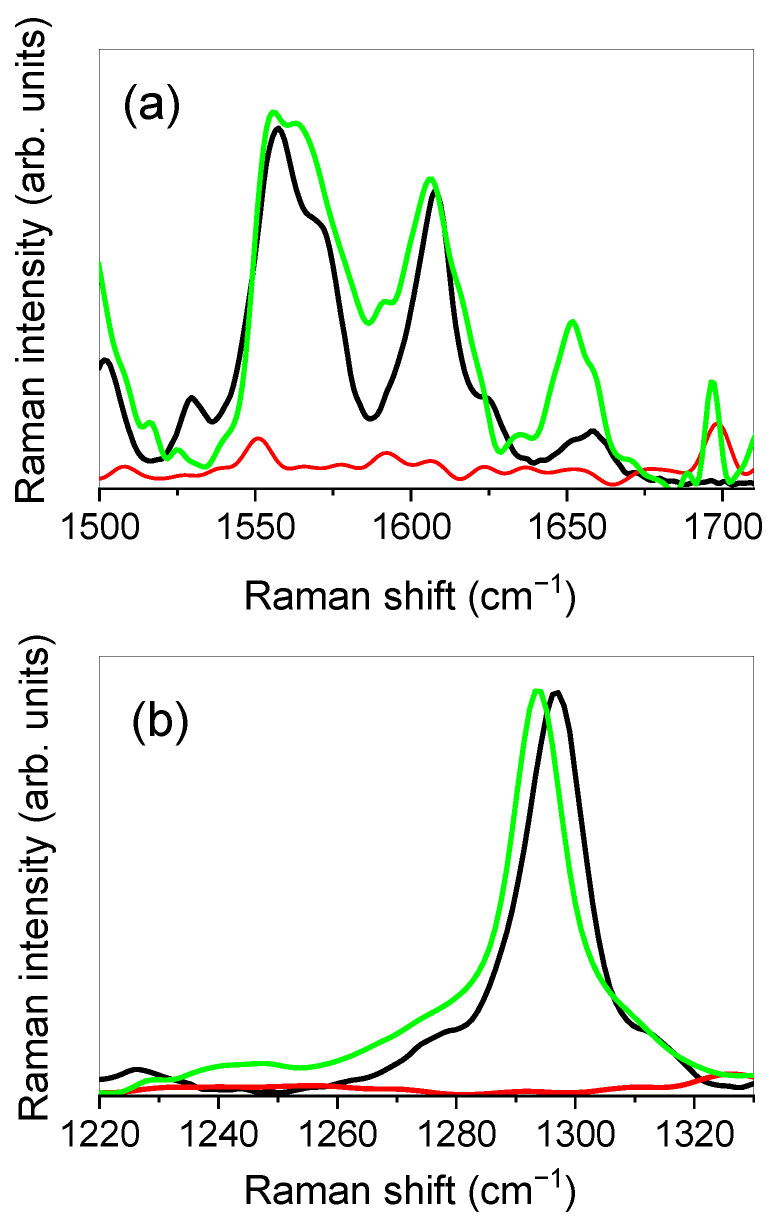
Experimental Raman spectra, respectively collected in the 1500–1710 cm^−1^ (**a**) 1220–1330 cm^−1^ (**b**) intervals, for RT (black line), SLNs (red line), and RT-SLNs (green line) systems. The negligible contribution of SLNs to the vibrational profile, in the investigated spectral range, is well evident.

Figure 4 depicts the experimental Raman spectra, in the 1500–1710 cm^−1^ (a) and 1220–1330 cm^−1^ (b) ranges respectively, for RT, SLNs, and RT-SLNs systems.

From the inspection of Figure 4a, a change in the relative intensities of the peaks that contribute to the band between ≈1530 cm^−1^ and ≈1590 cm^−1^, associated with C=C stretching vibrations of the benzene and dihydroxyphenyl rings of RT, is first of all revealed when the drug is encapsulated in the SLNs. A slight shift toward lower wavenumbers of the band centered at ≈1611 cm^−1^, associated with C=C stretching vibrations of the benzene, heterocyclic, and dihydroxyphenyl rings of RT, is also observed. Going on, the band at ≈1657 cm^−1^ of RT, corresponding to the C=O stretching vibration the heterocyclic ring, exhibits an evident shift toward the low wavenumbers (the center-frequency of the band moves to ≈1651 cm^−1^), as well as a clear change in shape when RT is encapsulated in SLNs.

The above-discussed spectral changes provide experimental evidence of the activation of drug–nanoparticle interactions involving, in particular, the aforementioned functional groups. In particular, the marked shift toward low wavenumbers of the C=O stretching band highlights a weakening of the carbonyl bond, suggesting an electrostatic environment of the C=O group that is more intense than the one experienced by the same group in the case of the pure drug. Therefore, this allows us to hypothesize, in agreement with previous studies on similar systems [61], a more intense association due to the establishment of new interactions.

Finally, interesting changes were also revealed in the lower frequency region, from 1220 to 1330 cm^−1^ (Figure 4b). It is clearly evident, as can be seen from the figure, a shift toward low wavenumbers of ≈5 cm^−1^ of the C–O and C–C stretching vibration band of the heterocyclic ring of RT, centered at ≈1297 cm^−1^ for the pure drug, after the drug encapsulation in SLNs. This spectral modification suggests, in analogy with what was observed for the C=O band, the involvement of the C–O and C–C functional groups in the formation of drug–nanoparticle interactions, together with a more structured environment that surrounds these groups, which contributes to weakening their dipole moment.

### 2.4. Biological Anti-Oxidant Assay

**Figure 5:**
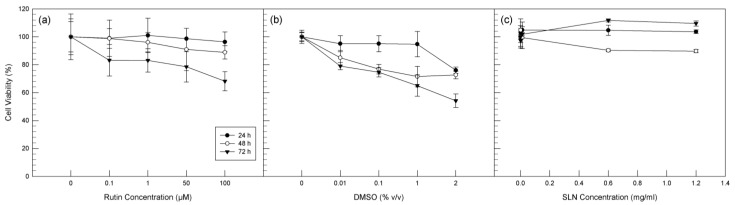
Effects on U373 cell viability of 24 h, 48 h, and 72 h treatment with different concentrations of free RT (**a**), DMSO (**b**), and blank SLNs (**c**). Cell viability was measured using the 3-(4,5-dimethylthiazol-2-yl)-2,5-diphenyltetrazolium bromide (MTT) test, and the results are presented as the mean of three different experiments ± standard deviation (S.D.). The error bar, if not shown, was within the symbol.

For in vitro test, the U373 cell line was used to test the anti-oxidant effect of RT in free form and, above all, to evaluate the ability of SLNs to improve the biological activity of the drug. As reported by other authors [62,63], RT is safety on U373 cells at low concentrations, but it shows anti-tumoral effects at high concentration. For this reason, before proceeding with lactic hydrogenase (LDH) assay to evaluate the anti-oxidant effects, 3-(4,5-dimethylthiazol-2-yl)-2,5-diphenyltetrazolium bromide (MTT) tests were carried out in order to evaluate the most suitable concentrations of RT to be used in order to maintain the anti-oxidant activity, preserving at the same time the cellular vitality. For this purpose, increased concentrations of free RT were tested, and the cytotoxic effects of free drug in terms of cell viability (%) were reported in Figure 5a. The treated cells began to suffer in the presence of high concentrations of RT and at the longest times tested, as already demonstrated [62,63]. In particular, a significant reduction (*p* < 0.05) of cell viability was recorded at 100 µM of free RT and after 72 h of exposure.

Since RT is a drug characterized by a very low solubility in water, for in vitro test, it was solubilized in dimethyl sulfoxide (DMSO). For this reason, it has been necessary to test the effect of solvent on cell viability and, therefore, U373 cells were treated with the same volumes of DMSO that was used to solubilize RT at increased concentrations. As can be seen from Figure 5b, when the cells are treated with DMSO alone at the same concentrations used for the in vitro tests with RT, they suffer a significant reduction in viability (*p* < 0.05) already after 48 h of treatment and at lower concentration (0.1% *v*/*v* DMSO). We considered these results interesting, since RT seems to reduce the cytotoxic effects induced by DMSO. Blank SLNs revealed no influence on U373 cells viability, as reported in Figure 5c. It is noteworthy that the blank SLNs concentrations reported in the figure correspond to the amount of RT-SLNs necessary to reach the selected concentration of RT in free form.

Based on these in vitro results, we decided to use 50 µM as a maximum concentration of RT in free form and as RT-SLNs for subsequent LDH assay.

To induce an oxidant stress, U373 cells were treated with hydrogen peroxide (H_2_O_2_) at 700 µM and for 1 h. This concentration and this incubation time were chosen based on the MTT test previously carried out on U373 cells in the presence of different H_2_O_2_ concentrations (300 µM–1 mM) for several incubation times (1, 2, and 3 h) (data not shown). 

**Figure 6:**
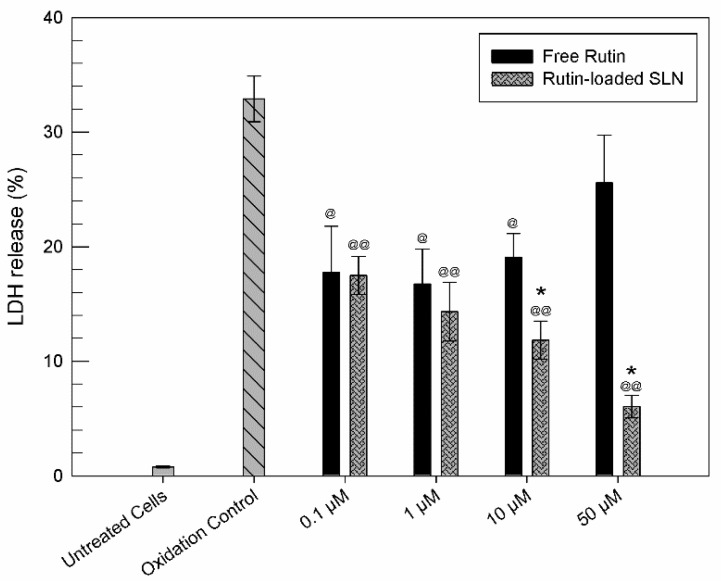
Anti-oxidant effect of RT and RT-SLNs on U373 cells expressed as LDH release reduction. Cells were treated with increased concentration of RT and RT-SLNs for 24 h and then incubated with H_2_O_2_ (700 µM) for 1 h. Results are presented as the mean of three different experiments ± S.D. The error bar, if not shown, was within the bar. The data obtained for RT-SLNs are statistically significant with respect to the same concentration of free RT (* *p* < 0.05), while the data obtained for RT and RT-SLNs are statistically significant with respect to oxidation control (@ *p* < 0.05; @@ *p* < 0.001).

As reported in Figure 6, the cells treatment with H_2_O_2_ caused a strong release of LDH (about 35%) with respect to untreated cells. The treatment of cells with free drug (0.1, 1, 10 µM) leads to a reduction of LDH release, with respect to oxidation control, so demonstrating the ability of RT to protect cells from the oxidizing effect of H_2_O_2_. The highest tested concentration of RT (50 µM) showed a reduced protective effect on U373 cells, which was probably because this concentration is close to the minimum RT concentration able to induce a cytotoxic effect, as demonstrated by MTT tests. As shown in Figure 6, the presence of SLNs permitted obtaining a reduction of LDH release, with respect to the same concentration of free drug. This occurrence testifies that the encapsulation of drug within SLNs allows enhancing the anti-oxidant activity of RT at all tested concentrations. This effect is particularly evident when cells are pre-treated with 10 and 50 µM of RT-SLNs.

This effect could be due to an unspecified *kiss-and-run* mechanism, as described by Hofmann et al. [64], in which the contact of nanoparticles with the cells produces the transfer of the drug from nanocarrier to cell membranes, increasing permeation. We should also consider the probable transport of RT inside the cells by means of a facilitated diffusion mediated by the GLUT2 transporter, as demonstrated by Zhang et al. [65]. The GLUT2 transporter is present in U373 cells, even if less expressed than GLUT1 [66]. The gradual release of RT from SLNs could avoid the saturation of the transporter, so facilitating its activity. Finally, an internalization of RT-SLNs by the cells could not be excluded [67,68]. If this is the case, the drug could be gradually released from the SLNs directly into the cytoplasm, and it does not need permeation through cell membranes to show its protective effects. In this sense, future studies are planned to confirm this hypothesis. On the other hand, no statistically significant anti-oxidant effects of empty SLN have been shown (Figure S1 Supplementary Material).

## 3. Materials and Methods

### 3.1. Materials

Phospholipon^®^ 80H is a Phospholipid GmbH (Cologne) product. Rutin hydrate (MW 610,2, RT), minimum 95%, Tween 80, trehalose from Saccharomyces Cerevisiae ≥ 99%, 3-[4,5-dimethylthiazol-2-yl]-3,5-diphenyltetrazolium bromide (MTT) dye test (TLC purity ≥97.5%), sodium dimethyl sulfoxide (DMSO) and phosphate buffer solution (PBS, pH 7.4) were purchased from Sigma Aldrich (Milan, Italy). Ethanol HPLC grade is a Merck^®^ product. Acetone HPLC grade was purchased from J.T. Baker^®^. Dulbecco’s modified Eagle’s medium (DMEM), heat-inactivated fetal bovine serum (FBS), penicillin (100 UI/mL)/streptomycin (100 µg/mL) solution, amphotericin B (250 μg/mL), glutamine, and trypsin/ethylenediaminetetraacetic acid (EDTA) (1×) solution were obtained from GIBCO (Invitrogen Corporation, Giuliano Milanese, Milano, Italy). Human glioblastoma astrocytoma cells (U373) were purchased from ATCC (American Type Culture Collection) retailer LGC Standards S.r.l. (Sesto San Giovanni, Milano, Italy). The Pierce lactic dehydrogenase (LDH) cytotoxicity assay kit was obtained from Thermo Fisher-Scientific (Waltham, MA, USA). The water used throughout the study was double-distilled, then filtered through 0.2 μm polytetrafluoroethylene (PTFE) filters (Millipore). Dialysis bags were Spectra/Por^®^ Dialysis Membrane (MWCO: 8000, Spectrum Laboratories, Inc., Rancho Dominguez, CA, USA).

### 3.2. Preparation of Blank SLNs and RT-SLNs

The blank SLNs were prepared by a solvent emulsification/diffusion method, as reported in the literature [25], by using the following phases: (i) aqueous phase, constituted by 22.5 mL water containing 365 mg Tween 80; (ii) a lipid phase, constituted by 100 mg Phospholipon^®^ 80H solubilized in 2.5 mL of 2:1 ratio ethanol/acetone mixture (*v*/*v*). Both solutions were heated at temperatures slightly higher than 60 °C. The lipid phase was added dropwise in the aqueous phase, during high-speed homogenization, using Ultra-Turrax T 25 (IKA-Werke, Staufen, Germany), at 11,000 rpm for 7 min. The homogenized suspension underwent ultrasonication (Bandelin Sonorex^®^ RK 514, Berlin, Germany) for 5 min to reduce the size of SLNs. Then, it was allowed to cool to room temperature under magnetic stirring (300 rpm) for 24 h to permit the evaporation of organic solvent and then centrifuged for 20 minutes at 5000 rpm (Heraeus Megafuge 16 centrifuge) to remove the lipid that did not interact. The supernatant was collected, added to 5% (*w*/*v*) trehalose as cryoprotectant and then freeze-dried (−53 °C, 43 mTorr) for 48 h (VirTis Gardiner, USA BenchTop K Series Freeze Dryers).

To prepare RT-SLNs, different concentrations of RT (2%, 5%, 8%, and 10%, *w*/*w*) were dissolved in the heated lipid under continuous magnetic stirring; then, the same preparation procedure described for blank SLNs was performed. After centrifugation for 20 min at 5000 rpm, the pellets containing the lipid and excess RT were collected and used for indirect determination of RT encapsulated into SLNs. The supernatant was added to 5% (*w*/*v*) trehalose as cryoprotectant and then freeze-dried for 48h.

### 3.3. Technological Characterization of RT-SLNs

Particle size (i.e., hydrodynamics radius, R_H_) and polydispersity index (PDI) of the RT-SLNs were determined by dynamic light scattering (DLS) technique using Zetamaster (Malvern software, submicron particles analyzer). Furthermore, zeta potential (ζ) was measured using ZetaPals Bookhaven Instruments Corporation.

The freeze-dried RT-SLNs were weighed and the yield percentage (yield (%)) was calculated using the following formula:

Yield (%) = (actual weight/theoretical weight) × 100.(1)

The entrapment efficiency percentage (E.E. (%)) and drug loading percentage (D.C. (%)) were indirectly determined on the pellets obtained after centrifugation at 5000 rpm of RT-SLNs dispersion. The pellets were added to cold ethanol (5 mL) that solubilized only RT. As Phospholipon^®^ 80H is soluble only in hot ethanol (~ 60 °C), it remained as a solid. The suspensions were filtered through 0.45 µm PTFE filters (Millipore®), the filtrates containing RT were collected and analyzed by UV-Vis spectroscopy (FullTech Instruments, Rome, mod. PG T80) at 259 nm, at 25.0 ± 0.1 °C, using 10.00 mm quartz cells (Hellma), to determine the amount of RT outside the SLNs. This procedure was repeated four times, until no active ingredient was detected, so guaranteeing the complete recovery of RT. The difference between the amount of initial RT and the free drug in the pellets represents the amount of drug loaded in the SLNs.

E.E. (%) and D.C. (%) were respectively calculated according to the following equations:

E.E. (%) = [(RT initially added−free RT outside SLNs)/RT initially added] × 100(2)

D.C. (%) = [(RT initially added−free RT outside SLNs)/recovered SLNs] × 100.(3)

### 3.4. In Vitro RT Release

The in vitro release study of RT from the SLNs was carried out by dialysis at 37 ± 0.5 °C by Telesystem 15.40 thermostated bath with a Telemodul 40 C control unit, in phosphate buffer solution (PBS, pH 7.4) and ethanol (15%). The dialysis bags were wet in PBS (pH 7.4) for 24 h before use.

Freeze-dried RT-SLNs samples (21 mg) were redispersed in 6 mL of PBS (pH 7.4); then, they were poured into the dialysis bag and placed under magnetic stirring (100 rpm, 37.0 ± 0.5 °C) into a beaker containing the dialysis medium (100 mL). At specific time intervals (30 min, 1, 3, 24, 48, and 72 h), the dialysis medium was removed and replenished with fresh PBS to maintain the original volume. All the collected volumes were evaporated under vacuum at 25.0 ± 0.1 °C, and the residues were solubilized in ethanol (2 mL) and analyzed by UV-vis spectroscopy at 259 nm. The experiments were done in triplicate and expressed as mean value ± S.D. The studies were conducted by comparing free RT.

The release data were treated according different kinetic models, i.e., zero-order (cumulative percentage of released drug vs. time), first-order (log cumulative percentage of remaining drug vs. time), Higuchi (cumulative percentage of released drug vs. square root of time), and Korsmayer–Peppas equations (log cumulative percentage of released drug vs. log time) [54].

### 3.5. Scanning Electron Microscopy

Scanning electron microscopy (SEM) characterization was performed by a Zeiss (Germany) Supra 25 Scanning Electron Microscope with a Schottky field emission gun. The acceleration voltage can be varied in the 0.1–30 kV range. RT-SLNs were analyzed in plan view by using an acceleration voltage of 2 kV. The RT-SLNs were spanned on a conductive substrate. The analysis was realized by using an in-lens detector.

Two different images at different magnifications were collected, as visible by different markers reported in the images. The acquired images were further analyzed by the software ImageJ obtaining the particle count and their dimension through the contrast difference in a binary converted image. The statistical analysis was further performed by Matlab (MathWorks, Inc., Natick, MA, USA).

### 3.6. Raman Spectroscopy

Raman measurements were performed by using a DXR-Smart Raman Spectrometer (Thermo Fisher Scientific). The experimental set-up was equipped with a Universal Platform Sampling (UPS) accessory. The spectra were acquired using an He-Ne laser source operating at 785 nm, with a power output of 24.0 mW and a full range grating of 400 lines/mm; all the spectra were recorded over the wavenumber range of 50–3300 cm^−1^, with a resolution of 1.9 cm^−1^.

The 180-degree sampling accessory was used to carry out the measurements, and two different sample holders were used. In particular, for SLNs and RT-SLNs, which originally appeared already in the form of freeze-dried pellets, a special sample holder was used; instead, RT and Phospholipon^®^ 80H, originally in the form of powders, and Tween 80, in the form of a viscous liquid, were allocated inside dedicated cuvettes.

In order to maximize the signal-to-noise ratio (S/R), 64 sample frames with an exposure time of 10 s, for a total acquisition time of 64 s per spectrum, were set before each acquisition.

### 3.7. Biological Assay

#### 3.7.1. Cell Cultures

Human glioblastoma astrocytoma (U373) cells were incubated using suitable plastic culture dishes (100 mm × 20 mm) in a Guaire^®^ TS Autoflow Codue Water-Jacketed incubator at 37 °C (5% CO_2_), in the presence of Dulbecco’s Modified Eagle Medium (DMEM) supplied by penicillin (100 UI/mL), streptomycin (100 μg/mL), amphotericin B (250 μg/mL), and fetal bovine serum (FBS) (10% *v*/*v*). The culture medium was replaced with fresh DMEM every 48 h. When ≈80% confluence of cells was reached, trypsin (2 mL) was used for cell detaching. The detached cells were collected into a centrifuge tube containing 4 mL of the fresh culture medium and centrifuged at room temperature for 5 min at 1200 rpm with an Eppendorf Centrifuge 5810. The obtained pellet was re-suspended with fresh medium, and cells were used for subsequent in vitro studies.

#### 3.7.2. Evaluation of In Vitro Cytotoxic Activity by MTT Assay

The U373 cells were placed in a 96-well plastic culture plate at a fixed density of 8000 cells/0.2 mL in triplicate. After 24 h of incubation time at 37 °C and 5% CO_2_, the culture medium was replaced with fresh DMEM medium, containing different concentration (0.1, 1, 50, 100 µM) of free RT, or blank SLN, or DMSO, followed by re-incubation for 24, 48, and 72 h. Eight wells for each plate were used as control with untreated U373 cells. After incubation time, 10 µL of MTT (5 mg/mL dissolved in PBS solution) were placed in each well; after 3 h of incubation, supernatants were removed and DMSO/ethanol solution (1:1 *v*/*v*, 100 μL) was added to each well to solubilize the colored formazan crystals. The cell viability (%) was determined by an ELISA microplate reader (xMark™ Microplate Absorbance Spectrophotometer, Bio-Rad, Italy) at λ_abs_ = 570 nm and λ_em_ = 670 nm, according to the following equation:

Cell Viability (%) = AbsT/AbsC × 100(4)

where AbsT and AbsC represent the absorbance of treated and untreated (control) cells, respectively. The results were reported as the mean value of three different experiments ± S.D.

#### 3.7.3. Determination of LDH Release for Anti-Oxidant Activity Evaluation

The anti-oxidant activity of free RT and RT-SLNs was evaluated by means of LDH assay, which is a method for the detection of membrane alteration and cell disruption [59]. The U373 cells were placed in 96-well plates and treated for 24 h with increased RT concentration (0.1, 1, 10, and 50 µM) in free form and loaded in SLNs; then, the cells were incubated with hydrogen peroxide (700 µM) for 1 h. The anti-oxidant activity of compounds was evaluated in terms of LDH release using a Pierce LDH cytotoxicity assay kit and following its specific protocol. LDH release was analyzed by spectrophotometer (xMark™ Microplate Absorbance Spectrophotometer, Bio-Rad, Milan, Italy) in the cultured medium at λ_abs_ = 680 nm and λ_abs_ = 490 nm. The results were reported as the mean value of three different experiments ± S.D.

#### 3.7.4. Statistical Analysis

One-way ANOVA testing was carried out to determine statistical significance. A Bonferroni t-test was used to validate the ANOVA test, and *p* < 0.05 was considered as statistically significant.

## 4. Conclusions

Rutin-loaded SLNs were formulated using a solvent emulsification–diffusion followed by sonication technique; then, they were characterized for particle size, zeta potential, drug entrapment, in vitro RT release, surface morphology, drug–nanoparticle interactions, and in vitro cytotoxic and anti-oxidant activity. DLS gave a mean size of particles in the range of 40–170 nm, and a zeta potential between −23 and −20 mV.

High E.E. (%) and D.C. (%) values were obtained. In vitro RT release studies demonstrated a sustained release of drug (about 50%) within 72 h. Data analysis allowed us to hypothesize Fickian diffusion as the main release mechanism. 

Morphological examination of RT-SLNs by SEM revealed spherical nanoparticles with a smoothed surface. Raman spectroscopy furnished evidence of new drug–nanoparticle interactions, involving the C=C groups of the benzene, heterocyclic, and dihydroxyphenyl rings, as well as the C=O group of the heterocyclic ring of RT.

RT-SLNs are shown to be safe for U373 cells and to produce a potent protective anti-oxidant effect at 50 μM of dose.

It is finally worth remarking that since RT is an herbal supplement, results on its efficacy can be contradictory, and many still unanswered questions remain. In the light of this, other biological experiments on different cell lines, as well as in vivo studies, are planned in order to support the obtained results.

## Figures and Tables

**Table 1:** Hydrodynamic radius, (R_H_), polydispersity index (PDI), zeta potential (ζ), yield (%), encapsulation parameters (encapsulation efficiency percentage (E.E. (%)), drug content percentage (D.C. (%))) of solid lipid nanoparticles loading rutin (RT-SLNs) prepared using different theoretical amount of RT. n.d. = not detected.

RT (%)	R_H_ (nm)	PDI (%)	ζ (mV)	Yield (%)	E.E. (%) ± S.D.	D.C. (%) ± S.D.
2	40 ± 0.5	<30	−23 ± 0.05	96 ± 5	97 ± 2	1.9 ± 0.5
5	60 ± 0.3	<30	−21 ± 0.03	95 ± 3	98 ± 1	4.6 ± 0.3
8	80 ± 2.0	>30	−21 ± 0.08	92 ± 6	n.d.	n.d.
10	170 ± 4.4	>30	−20 ± 0.06	90 ± 2	n.d.	n.d.

**Table 2:** Regression coefficient (R^2^), rate constant (K_i_, i = 0 for zero-order, 1 for first-order, and H for Higuchi model, respectively), and transport exponent (n) of Korsmeyer–Peppas model, as obtained from release data of RT from SLNs.

Zero-Order Model		First-Order Model		Higuchi Model		Korsmayer–Peppas Model	
R^2^	K_0_ (h^−1^)	R^2^	K_1_ (h^−1^)	R^2^	K_H_ (h^−1/2^)	R^2^	N
0.8224	0.5227	0.8656	0.0077	0.9344	5.0548	0.8837	0.4282

## Data Availability

The data presented in this study are contained within the article and supplementary material.

## Supplementary Materials

The following are available online. Figure S1: Anti-oxidant effect of empty SLN on U373 cells expressed as LDH release reduction. Cells were treated with increased concentration of empty SLN for 24 h, then incubated with H2O2 (700 µM) for 1 h. Noteworthy, the empty SLNs concentrations reported in the figure correspond to the amount of RT-SLNs necessary to reach the selected concentration of RT in free form. Results are presented as the mean of three different experiments ± S.D. The error bar, if not shown, was within the bar. The data obtained for empty SLN are not statistically significant with respect to the oxidation control.

## Sample Availability

Not available.
