# Supplementary material for: Rutin-Loaded Solid Lipid Nanoparticles: Characterization and In Vitro Evaluation
Source: Molecules. 2021 Feb 16;26(4):1039. doi: 10.3390/molecules26041039 (PMC7920302; doi:10.3390/molecules26041039)
Supplement: Supplementary file 1 [file molecules-26-01039-s001.pdf]

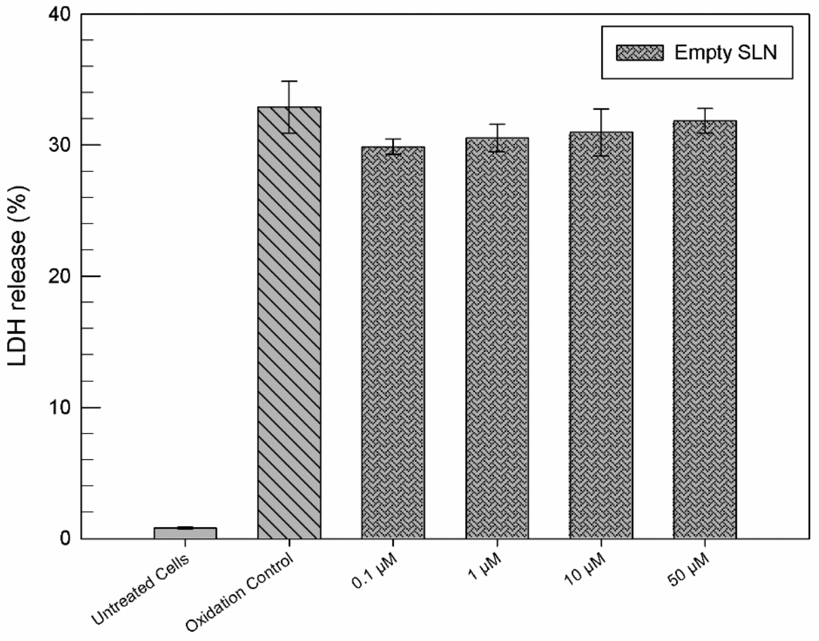

Figure S1: Anti-oxidant effect of empty SLN on U373 cells expressed as LDH release reduction. Cells were treated with increased concentration of empty SLN for 24h, then incubated with H_2O_2 (700 µM) for 1 h. Noteworthy, the empty SLNs concentrations reported in the figure correspond to the amount of RT-SLNs necessary to reach the selected concentration of RT in free form. Results are presented as the mean of three different experiments ± S.D. The error bar, if not shown, was within the bar. The data obtained for empty SLN are not statistically significant with respect to the oxidation control.
